# Low Susceptibility of Invasive Red Lionfish (*Pterois volitans*) to a Generalist Ectoparasite in Both Its Introduced and Native Ranges

**DOI:** 10.1371/journal.pone.0095854

**Published:** 2014-05-05

**Authors:** Paul C. Sikkel, Lillian J. Tuttle, Katherine Cure, Ann Marie Coile, Mark A. Hixon

**Affiliations:** 1 Department of Biological Sciences, Arkansas State University, Jonesboro, Arkansas, United States of America; 2 Department of Integrative Biology, Oregon State University, Corvallis, Oregon, United States of America; 3 The Marine Laboratory, University of Guam, Mangilao, Guam; 4 School of Plant Biology and Oceans Institute, The University of Western Australia, Crawley, Australia; 5 Department of Biology, University of Hawai'i at Mānoa, Honolulu, Hawai'i, United States of America; College of Charleston, United States of America

## Abstract

Escape from parasites in their native range is one of many mechanisms that can contribute to the success of an invasive species. Gnathiid isopods are blood-feeding ectoparasites that infest a wide range of fish hosts, mostly in coral reef habitats. They are ecologically similar to terrestrial ticks, with the ability to transmit blood-borne parasites and cause damage or even death to heavily infected hosts. Therefore, being highly resistant or highly susceptible to gnathiids can have significant fitness consequences for reef-associated fishes. Indo-Pacific red lionfish (*Pterois volitans*) have invaded coastal habitats of the western tropical and subtropical Atlantic and Caribbean regions. We assessed the susceptibility of red lionfish to parasitic gnathiid isopods in both their native Pacific and introduced Atlantic ranges via experimental field studies during which lionfish and other, ecologically-similar reef fishes were caged and exposed to gnathiid infestation on shallow coral reefs. Lionfish in both ranges had very few gnathiids when compared with other species, suggesting that lionfish are not highly susceptible to infestation by generalist ectoparasitic gnathiids. While this pattern implies that release from gnathiid infestation is unlikely to contribute to the success of lionfish as invaders, it does suggest that in environments with high gnathiid densities, lionfish may have an advantage over species that are more susceptible to gnathiids. Also, because lionfish are not completely resistant to gnathiids, our results suggest that lionfish could possibly have transported blood parasites between their native Pacific and invaded Atlantic ranges.

## Introduction

Parasites make up about 40% of the earth's biodiversity and parasitism constitutes the most common type of ecological interaction [1]. Because of their effects on host population dynamics, parasites directly or indirectly influence the dynamics and structure of ecological communities [2]. One aspect of host-parasite ecology that has received considerable recent attention is the extent to which host-parasite interactions both influence and are influenced by the spread of invasive species [3], [2].

Parasites can influence the success of introduced species relative to native species in multiple ways. The effects of host-specialists are easiest to predict and have received the most attention as components of “enemy release” [4], [5]: introduced species likely leave behind specialist parasites from their native range and are likely avoided, at least initially, by specialist parasites in the introduced range due to a lack of shared evolutionary history. The dynamics involving introduced hosts and generalist parasites are more difficult to predict. Exotic hosts may introduce generalist parasites to which they have high resistance but to which native species have limited or no resistance [6], [7]. Invasive species may also be more or less susceptible to generalist parasites in the introduced range, influencing the dynamics between hosts and parasites as well as the transmission of any disease-causing organisms transmitted by generalist parasites [8], [9], [10]. For example, one longitudinal study discovered that ticks more heavily infest introduced chipmunks than native voles [11]. However, some other studies of wild-caught hosts revealed that non-native species tend to have fewer generalist parasites (external crustaceans and internal helminths) than their sympatric, native counterparts [12], [13].

Gnathiid isopods (Crustacea) are generalist ectoparasites of fish hosts in both temperate and tropical oceans [14], [15]. They are unusual among parasites of fishes in that only the larvae are parasitic (protelean parasitism). The larval phase consists of three stages (instars), ranging from approximately 0.5–3 mm in length that emerge from the substratum mostly at night and dawn and find host fish. When engorged on blood and body fluids, they return to the substratum and molt into the next larval stage. After the final blood meal, third stage larvae metamorphose into adult males or females that live in the benthos and do not feed [14], [15].

Gnathiids are of particular ecological importance on tropical reefs where they are an abundant crustacean parasite infesting a wide range of fish hosts [16], [17], [18]. They transmit protozoan blood parasites [19], [20], [21], reduce hematocrit [22] and can even be a direct cause of death in heavily-infected and/or small hosts [23], [24], [25]. Gnathiids are also the primary food item of cleaner mutualists such as cleaner wrasses in the Indo-Pacific [26] and cleaner gobies in the Caribbean [27], [28] and they influence the time hosts spend interacting with cleaning organisms [29], [30]. At sites on the Great Barrier Reef, removal of the cleaner wrasse *Labroides dimidiatus*, a main predator of gnathiids, has been shown to reduce growth and abundance of at least one host species [31]. Thus, the degree of susceptibility to gnathiids can have significant fitness consequences for reef-associated fishes, and can therefore influence the success of an introduced species in the coral-reef ecosystem.

While various species of marine fishes have been introduced by human activities to new habitats worldwide, our understanding of the role of parasites in these invasions lags far behind freshwater systems [32]. One marine fish in particular, the Indo-Pacific red lionfish (*Pterois volitans*), was listed in a recent review as one of the top 15 global conservation issues [33]. Since their accidental or intentional release from aquaria off the coast of Florida in the mid-1980s, lionfish have undergone a population explosion and now range throughout the western tropical and subtropical Atlantic, Caribbean, and Gulf of Mexico [34], [35]. The invasion has significantly affected native coral-reef ecosystems, mainly via predation of a broad diversity of juvenile fishes and crustaceans [36], [37], causing substantial reductions in the recruitment and abundance of reef fishes [38], [39], [40] and also negatively affecting native piscivorous predators via both predation of juveniles and possible competition with adults [41].

Greater local density (>390 fish/ha vs. 6 fish/ha) and maximum size (45 cm total length TL vs 38 cm TL) of lionfish in the invaded Atlantic relative to their native Pacific [42], [43] suggests some level of ecological release from natural control mechanisms, such as competition, predation, and parasitism [43], [44], [45], and the apparent inability of native reef communities to provide natural enemies that can offset invading lionfish indicates a lack of biotic resistance to the invasion [46].

The goal of our study was to test the susceptibility of lionfish to parasitic gnathiid isopods at sites in both their native Pacific and introduced Atlantic ranges, relative to other species and under natural conditions. Disentangling the effects of exposure of hosts due to differences in encounter rates and other factors that influence the probability of infestation is essential for understanding variation in the susceptibility of hosts to ectoparasites. Most studies of components of host susceptibility to ectoparasites in terrestrial [47], [48], [49], [50], [51] and aquatic environments [52], [53], [54], [55], [56], [57] are based solely on either cultured, laboratory-reared parasites, or are observational studies of parasites found on wild-caught hosts. While these studies are instructive, they do not control for differences in host habitat utilization, and often fail to control for temporal variation in the activity of ectoparasites. To our knowledge, ours is the first field experimental study to compare the susceptibility to generalist parasites of invasive and native hosts in either the invaded or native range.

## Materials and Methods

### Ethics statement

Permits to conduct this field study were obtained from the Bahamian Department of Marine Resources, the Cayman Islands Marine Conservation Board, the Virgin Islands National Park, and the Philippines Department of Agriculture, Bureau of Fisheries and Aquatic Resources. No protected species were used. All methods were consistent and compliant with approved guidelines for the treatment of fishes in a research capacity by the Arkansas State University and Oregon State University Institutional Animal Care and Use Committees (IACUC), and the Oregon State University IACUC specifically approved this study (ACUP number 3886). To minimize stress, fish were held in flow-through aquaria for no more than one day before deployment in experimental cages. After fish were retrieved from the reef, they were cleaned of all external parasites and allowed to recover in aerated seawater for a 2–3 hours before being either released into the wild (for native fishes), or in the case of invasive lionfish, humanely euthanized by quickly severing the spinal cord.

### Introduced range of lionfish

#### Field and laboratory methods

The subtropical western Atlantic and Caribbean (introduced range) component of this study was conducted on nearshore reefs off Lee Stocking Island, Bahamas (23°46′00″N, 76°06′00″W), and Little Cayman, Cayman Islands (19°41′56″N, 80°3′38″W), during the summers of 2009 and 2010, respectively, and off St. Thomas (18°20′00″N, 64°50′00″W) and St. John (18°19′00″N, 65°44′00″W), US Virgin Islands, and Guana Island, British Virgin Islands (18°30′00″N, 64°38′00″W) in 2011–2012. We compared gnathiid loads between invasive lionfish, and native grunt species (*Haemulon plumierii* in the Bahamas, and *H. flavolineatum* in the Cayman Islands and Virgin Islands). Grunts were used as comparison species because they are abundant on shallow reefs throughout the Caribbean region, easy to catch, and comparable in size to lionfish. Most importantly, they are known to be infested by gnathiids in the Caribbean [58] and thus are a sensitive natural “assay” for the presence of gnathiids, which are known to vary considerably in abundance within and among sites [17], [59], [60], [61]. Because of these characteristics, grunts have been used as a “standard” for comparison among multiple native Caribbean species [62], enabling a comparison of lionfish with some other species as well (see below).

Field methodology followed Sikkel et al. [17], [18] and Coile and Sikkel [62]. In all experiments, no individual fish was used more than once. Grunts were collected on SCUBA from nearby reefs by trapping them in hand nets (Bahamas and Cayman Islands), or by free divers using modified casting nets (Virgin Islands). After capture, the live fish were placed unharmed into a large 20-liter plastic collecting bag or bucket until the end of the collecting period, and then transferred to a 1.5×0.25×0.30 m insulated container of aerated seawater for transportation to the field station. Collection took place between 08:00 and 13:00 h (when gnathiid infestation rates are typically low, [17]) to minimize the chance of gnathiid infestation prior to deployment of fish back to the reef. After collection, the fish were kept in shaded, aerated tanks for 5–8 hours, further allowing any gnathiids that were on the fish to complete feeding and dislodge. The majority of lionfish were collected in the same way. Additional lionfish (Virgin Islands only) were obtained from a local fisherman who removed live fish from his fish traps.

For each replicate series, fish were placed individually in cages, approximately 30 cm in diameter ×48 cm in length, constructed of black, 1.5 cm square mesh hardware cloth (“Vexar”). Each fish was visually inspected for gnathiids prior to being placed in a cage. Cages were interspersed in shallow (<10 m depth) reef habitat at dusk, secured with a lead diving weight. During any given dusk deployment, at the Bahamas and Virgin Islands sites, each lionfish was placed within 1–2 m of at least one native grunt comparison fish, and 2–8 m from another lionfish, each fish in its own cage. At the Cayman Islands sites, lionfish and grunts were placed in pairs, separated by approximately 0.5 m, and pairs were separated by up to 10 m from each other. In the Virgin Islands and Cayman Islands, cages were further surrounded by plastic lattice (Virgin Islands) or placed inside wire fish cages (Cayman Islands) to prevent predation by sharks, eels, and groupers. Reef locations were chosen that were structurally complex (dead and live coral, sponge, algae, rock) and therefore likely to have gnathiids, and had no apparent cleaning station (i.e., cleaning gobies or shrimps) within a 5 m radius of any cage. Cages were retrieved the following morning between first light and sunrise. Thus, fish were exposed to gnathiids during the nocturnal and dawn peaks in activity [17], [18]. At the Bahamas study site, 12 fish (6 *P. volitans*, 6 *H. plumierii*) were deployed in each of two replicate series, one on 18–19 June and one on 21–22 June 2009. At the Cayman Islands study site, 10 fish (5 *P. volitans*, 5 *H. flavolineatum*) were deployed in each of three replicate series from 23 to 26 August 2010. In the Virgin Islands, a combined 16 *P. volitans*, and 18 *H. flavolineatum* were deployed in Brewers Bay, St. Thomas, over two replicate series from 15 May to 25 June 2011. A combined 11 *P. volitans* and 24 *H. flavolineatum* were deployed in Lameshur Bay, St. John, over two replicate series from 31 May to 2 July 2011 (additional grunts were used as part of another experiment). A single *P. volitans* was similarly deployed in White Bay, Guana Island, along with 5. *H. flavolineatum*. Where replicate series were run within the same site, cages were placed at different locations within the site during different replicate series.

Upon being retrieved from the reef, fish were brought slowly to the surface, and immediately placed in individual 11-liter buckets filled with clean, aerated seawater. Buckets were transported back to the field station and allowed to sit for 3 h to allow attached gnathiids to feed and dislodge from the host. Fish were removed from buckets, thoroughly rinsed with a squirt bottle, and gnathiids were filtered from the buckets using 55-µm plankton mesh. The number of gnathiids was then counted under a dissecting scope and recorded for each fish.

#### Statistical analyses

All analyses were performed using SYSTAT 13. Because of the overall low numbers of gnathiids found on any of the potential host species from the Bahamas and Cayman Islands sites, and the large number of zero values, we compared the prevalence (presence or absence) of gnathiids on lionfish versus grunts using a Fisher Exact Test. The low proportion of lionfish infested with gnathiids precluded further statistical comparison of the intensity of infestation (the number of gnathiids infesting a host).

Infestation by gnathiids was much higher at the Virgin Islands sites. Thus, we compared the density of gnathiids (number of gnathiids/fish surface area) on lionfish and grunts using a General Linear Model (GLM) with bootstrap resampling. Replicate series/site was initially used as a blocking factor in the analysis but was subsequently removed from the model after finding no significant effect of replicate series or the interaction between it and species (both p>0.20). Fish surface areas were calculated from tracings of the fish's body and fins using Image J [63] as described in Coile and Sikkel [62].

In order to compare the level of infestation of lionfish with other Caribbean reef fishes, data from the Virgin Islands sites were also compared with data from Coile and Sikkel [62] who used the same field methodology described in this study to compare susceptibility among 14 species (from 8 families and 3 orders) of native Caribbean reef fishes to gnathiid isopods in Lameshur Bay, St. John, Virgin Islands, during summer 2010 and 2011 (the same year our Virgin Islands lionfish data were collected). In order to control for spatio-temporal differences in gnathiid abundance and therefore allow for comparison between species tested at different times (it was impossible to test all species simultaneously), the average density of gnathiids on the 5 caged French grunt placed closest to each replicate fish was used as one covariate. To control for differences in the size of the “target” presented by each fish, its surface area was also used as a second covariate. This approach detected statistically significant differences in susceptibility to gnathiids among the native Caribbean species tested. We therefore used a similar approach to statistically compare infestation of gnathiids on lionfish in this study with five native species from Coile and Sikkel [62] that overlap in diet and habitat with lionfish. These include one large grunt species (*Haemulon sciurus*: Haemulidae n = 22), two species of snapper (*Lutjanus apodus*, n = 27 and *L. synagris*, n = 14: Lutjanidae), one grouper species (*Epinephalus guttatus*: Serranidae, n = 14), and one species of squirrelfish (*Holocentrus rufus*: Holocentridae, n = 19). To increase the sample size for native carnivorous species, we also included data from White Bay, Guana Island, in 2010 and 2011 (obtained using the same procedure) for *H. sciurus* (n = 2), *L. apodus* (n = 2), *E. guttatus* (n = 12), and *H. rufus* (n = 13), and for the squirrelfish *Holocentrus adscensionis* (Holocentridae) and the scorpionfish *Scorpaena plumieri* (Scorpaenidae) from both Lameshur Bay (n = 6 and 5, respectively), and White Bay (n = 16 and 2, respectively) also collected in 2010–2011.

We compared the number of gnathiids on these carnivorous native species with the number found on lionfish using a GLM with bootstrap resampling. As in the Coile and Sikkel study [62], fish host surface area and average density of gnathiids on 5 French grunt standards were used as covariates.

### Native range of lionfish

#### Field methods

The Philippines (native range) experiments were conducted between 4 and 22 August 2011 off Silliman Beach (9°19′46″N, 123°18′43″E) on the southeastern side of Negros, Philippines, using the methods described above for the Caribbean sites. Cages (8–10 per series) were placed on a shallow reef at dusk and retrieved at dawn over four different sampling series during this period. Lionfish (n = 16 total) were collected by divers using hand nets off Silliman Beach and Malatapay (9°6′2.50″N, 123°12′22.05″E). For this experiment, native reef fishes from three families that overlap with lionfish in diet and habitat were used for comparison. Fishes from these families were also included in the western Atlantic/Caribbean comparison. These fishes were obtained opportunistically from local fishers off Silliman Beach, as required by permit, except for 8 fish that were obtained from captive stock. Comparison species included two grunt species (*Diagramma pictum*, n = 3 captive and 3 wild and *Plectorhinchus lessonii*, n = 1 captive and 2 wild: Haemulidae), two snapper species (*Lutjanus lutjanus*, n = 5 wild and *L. kasmira*, n = 2 wild: Lutjanidae), and one grouper species (*Epinephalus ongus*, n = 4 captive and 2 wild: Serranidae).

#### Statistical analyses

Data were combined among species within the same family, and compared with lionfish using a GLM with bootstrap resampling. Unlike the previous comparisons in the Atlantic region, an initial analysis revealed no significant relationship with gnathiid loads and host surface area (probably because of overall lower gnathiid loads compared with Virgin Islands sites), and so this covariable was excluded from the model.

## Results

### Introduced range of lionfish

At the Bahamas site, all 13 grunts were infested with gnathiids (range  = 1–8, mean intensity  = 3, SD = 2.45), compared with only 1 of 11 lionfish (9.1% - infested with 1 gnathiid). This inter-species difference was highly significant (*Fisher Exact Test, p<0.001*). Similarly, at the Cayman Islands site, 12 of 15 (80.0%) grunts were infested (range  = 1–23, mean intensity  = 6, SD = 6), compared with only 4 of 15 lionfish (26.7% - infested with 1–3 gnathiids each). This difference was also highly significant (*Fisher Exact Test, p = 0.009*).

**Figure 1:**
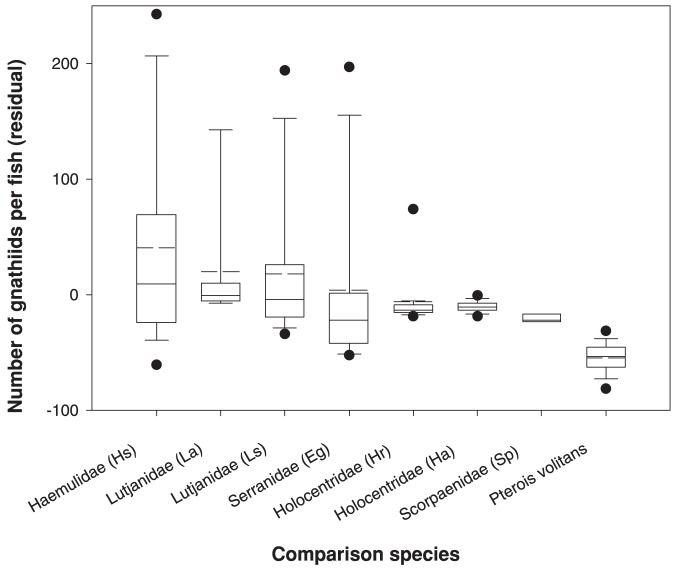
Box and whisker plot of infestation of gnathiid isopods on red lionfish, *Pterois volitans*, compared with ecologically similar, native tropical western Atlantic species. Levels of infestation are expressed as residuals from regression of gnathiid loads against host fish surface area and the local abundance of gnathiids as measured by the average gnathiid density on 5 French grunt “standards” placed near each host fish. Data for Caribbean species are grouped by families, with species codes given in parentheses: Haemulidae: Hs =  *Haemulon sciurus* (n = 24); Lutjanidae: La =  *Lutjanus apodus* (n = 29), Ls = *L. synagris* (n = 14); Serranidae: Eg =  *Epinephelus guttatus* (n = 26); Holocentridae: Hr =  *Holocentrus rufus* (n = 32); Ha =  *Holocentrus adscensionis* (n = 20); Scorpaenidae: Sp =  *Scorpaena plumieri* (n = 7); *Pterois volitans* (n = 30). Dashed line  =  mean, solid line  =  median, outliers are shown as single points.

At the Virgin Islands sites, 34 of 42 grunts (81.0%) were infested with gnathiids (range  = 1–222, mean intensity  = 57.68, SD = 71.58), compared with 12 of 28 lionfish (42.9%; range  = 1–23, mean intensity  = 5.92, SD = 6.36). Overall, lionfish had significantly fewer gnathiids than French grunt (*F_1,62_ = 5.24, p = 0.026*). When compared with 7 other ecologically similar Caribbean species at the Virgin Islands sites, lionfish also had the lowest gnathiid loads (*F = 10.88, df = 7,168, p<0.001*; Fig. 1).

### Native range of lionfish

**Figure 2:**
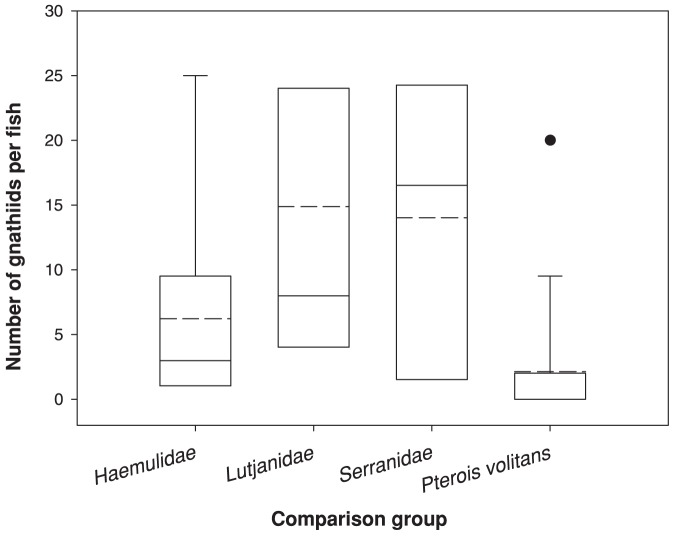
Box and whisker plot of the number of gnathiids on lionfish (n = 16) off Silliman Beach, Negros, Philippines, in comparison to native species of three other families of carnivorous reef fish (n≥6). Dashed line  =  mean, solid line  =  median, outliers are shown as single points. Note that median for lionfish is zero.

Less than half (43.8%) of the 16 lionfish had gnathiids following dawn retrieval. Six of these had five or fewer, although one had 20 (range  = 1–20, mean intensity 4.86, SD = 6.81). In contrast, 21 of the 22 (95.5%) individuals among comparison species had gnathiids (range  = 1–50, mean intensity  = 11.32, SD = 12.21, Fig. 2). Overall gnathiid loads differed significantly among species (*F = 5.37, df = 3,33, p = 0.004*), with lionfish being lowest (Fig. 2).

## Discussion

At two invaded localities (the Bahamas and Cayman Islands), most red lionfish did not become infested with gnathiids, whereas most individuals of the native comparison species (haemulid grunts) did. This result could be due in part to the apparently low overall gnathiid abundance at those sites. However, even when the experiment was repeated at invaded sites with much higher abundances of gnathiids (Virgin Islands), the results were similar; a higher proportion of lionfish became infested with at least one gnathiid, but densities were far lower than on native grunts.

Two interpretations of these results are that gnathiids at these sites have a narrow range of hosts they can infest, and/or that grunts are otherwise particularly susceptible to infestation. We did not identify the species of gnathiid from hosts at the Cayman Islands site. However, at the Bahamas and Virgin Islands sites, we identified gnathiids as *Gnathia marleyi* [64], which has thus far been shown to feed on blood from 19 different Caribbean fish species. Among the 14 Caribbean fish species (from 8 families and 3 orders) compared by Coile and Sikkel [62] in the Virgin Islands, haemulid grunts and lutjanid snappers were the most susceptible to gnathiid infestation. By employing the same protocol as that study, using French grunts as a standard, allowed us to compare our results for lionfish with samples from their study and thus compare the susceptibility of lionfish to that of multiple native Caribbean species. Although we limited statistical comparison in this study to carnivorous species that share habitat with invasive lionfish (i.e., controlling for covariables), gnathiid loads for lionfish deployed in Lameshur Bay, St. John (where Coile and Sikkel [62] conducted their study) were also lower than all 14 native species examined in that study.

Combined, these results suggest that lionfish are not highly susceptible to infestation by generalist ectoparasitic gnathiids in the Atlantic. Is this resistance limited to Atlantic gnathiids, or even to a particular species of Atlantic gnathiid, or are lionfish relatively resistant to gnathiids in general? Our results from identical experiments in the native range of lionfish (the Philippines) are consistent with the latter hypothesis. Although our sampling in the native range was limited to one site (we also conducted a single preliminary trial of the experiment at a site in Guam, but no gnathiids were found on any host) and fewer comparison species (limited by permit restrictions), lionfish also had the lowest gnathiid loads among comparison species.

To our knowledge, there is only one published account of gnathiids infecting *Pterois* spp., which comes from the eastern coast of South Africa where 2 individuals of *Gnathia pilosus* were found infecting a single adult devil firefish, *Pterois miles* [65]. *P. miles* and *P. volitans* are sibling species and both inhabit the western Atlantic, although *P. miles* appears to be limited to the southeast coast of the United States [35]. Furthermore, cleaning symbiosis experts in the Pacific and Indian Oceans report that lionfish rarely visit cleaning stations (A. Grutter and R. Bshary, personal communications, respectively). Considering that gnathiids are a primary food source for cleaner species [27], [66], and that gnathiid burden is positively associated with host interaction with cleaners [29], [30], low visitation rates by lionfish to cleaning stations could be explained, at least in part, by low gnathiid loads in their native range.

The mechanism by which lionfish appear to be less susceptible to gnathiids is currently unknown. However, there are multiple mechanisms by which teleost fish may reduce their susceptibility to external infection that may vary among some combination of taxa, localities, and individuals. For example, fish hosts may secrete excess mucosal epithelium [67], shed their scales [68], or initiate coagulation in response to ectoparasite attachment [69]. Skin toxins may also affect the attachment of ectoparasites on fishes [70]. Lionfish do possess venom in the grooves of their dorsal, pelvic, and anal spines that likely deter predation by larger fishes [71], [72]. While the venom itself is not located on the skin or in the blood, the venom precursor is systemic (Wilcox and Hixon in revision). It would be worthwhile to determine whether this chemical deters ectoparasites.

Lionfish in the subtropical and tropical western Atlantic appear to have spread from Florida from introduced individuals native to the Philippines and Indonesia [73]. Although our findings in the Atlantic appear robust among sites, and consistent with our experiment at one native locality, it is conceivable that invasive lionfish from a different population and/or native or invasive lionfish at other localities might exhibit higher susceptibility. Furthermore, while our comparison species represented the most common ecologically similar and sympatric species, these species were from different taxonomic families, with one exception (see below). It is possible that lionfish may appear relatively more susceptible if they are compared with more phylogenetically similar species. The fact that a native tropical western Atlantic scorpionfish (*S. plumieri*), a sedentary species from the same family as lionfish, also exhibited relatively low susceptibility compared with other species may provide insights. While this is the most common Caribbean and tropical western Atlantic scorpaenid [74], it is nowhere locally abundant, and we found no other genera of scorpaenid fishes at our Philippines site.

By using confined fish deployed in the field during the period of peak gnathiid activity, we found that both native and invasive red lionfish do not appear highly susceptible to generalist blood-feeding gnathiid isopods, compared with other common species in either their native Pacific or introduced Atlantic range. Previous studies that compared the generalist parasites infecting introduced vs. native host species have been observational [11], [12], [13], or conducted in the laboratory [12], and therefore cannot separate the relative contributions of exposure and susceptibility. While our study system is ecologically most similar to the tick-rodent system studied by Pisanu et al. [11], our results align more closely with Miller et al. [12] and Gendron et al. [13] where non-native species tended to have fewer generalist parasites (external crustaceans and internal helminths, respectively) than their sympatric, native counterparts. Time since introduction may also play a part in the relative susceptibility of invasive species to generalist parasites. Gendron et al. [13] revealed that more recently established populations of the invasive round goby (*Neogobius menalostomus*) in the Great Lakes-St. Lawrence River basin have fewer generalist helminths than older populations of invasive round goby. However, a study investigating the parasite fauna of peacock grouper (*Cephalopholis argus*) 50 years after their introduction to the Hawaiian archipelago, found that this invasive marine fish remains largely uninfected by native generalist and ubiquitous parasites [75]. Furthermore, in a study conducted almost 40 years after the appearance of rabbitfishes (*Siganus rivulatus* and *S. luridus*) off the Mediterranean coast of northern Africa, only eight cymothoid isopod individuals (generalist ectoparasites) were found on 375 rabbitfish [76]. Further studies should assess whether this pattern will exist in the decades following the establishment of invasive lionfish in the western Atlantic.

Considerable evidence suggests that introduced species tend to arrive in their new environments free from their natural parasites [3], [77], [78], which may enable them to more effectively establish and spread in their introduced habitat. However, generalist parasites might be able to exploit invasive species [79], which could reduce the fitness of infected individuals, thereby negatively affecting the invasive species population [80]. Our results demonstrate that lionfish are not highly susceptible to infestation by the most common and damaging generalist ectoparasite on coral reefs. The relatively low numbers of gnathiids infesting lionfish in both their native and invaded ranges suggest that these ectoparasites either are not highly attracted to or cannot effectively exploit lionfish as a host, and therefore cannot be expected to negatively affect populations of invasive lionfish. However, given that the susceptibility of lionfish to gnathiids is not zero, and that gnathiids transmit blood parasites, the possibility remains that, during the initial phases of their invasion, lionfish may have been vectors for blood parasites from their native range. Although fish imported through the aquarium trade are often treated for parasites before shipment and/or upon arrival, such treatments are applied inconsistently, depending on the collector or importer, and target external parasites. Indeed, protozoan blood parasites have been found in lionfish collected from some Atlantic localities [81]. Thus, future studies of parasites in lionfish should include assays for blood parasites.
